# Ongoing EEG delta and alpha rhythms reflect abnormal transitions from quiet wakefulness to sleep in patients with mild cognitive impairment due to AD and other diseases

**DOI:** 10.1002/alz70856_097703

**Published:** 2025-12-24

**Authors:** Veronica Henao Isaza

**Affiliations:** ^1^ Sapienza University of Rome, Rome, Rome, Italy; Universidad de Antioquia, Medellín, Antioquia, Colombia

## Abstract

**Background:**

Alzheimer's disease patients with mild cognitive impairment (AD) and mild cognitive impairment not due to Alzheimer's disease (MCI) exhibit distinct resting‐state eyes‐closed electroencephalographic (rsEEG) alpha rhythms (8‐12 Hz) and may show variations in daytime vigilance.

**Objectives:**

Our study compared EEG patterns during transitions from wakefulness to light sleep across AD, MCI, and healthy elderly (Nold) groups.

**Methods:**

EEG recordings of approximately 30 minutes included eyes‐open, quiet wakefulness, and light sleep stages. Frontal and posterior cortical activities were evaluated across delta and alpha frequency bands using ANOVA models adjusted for age and education.

**Results:**

Compared to Nold, AD participants exhibited significantly greater frontal delta source activities during wakefulness (*p* = 0.002) and ripples (*p* < 0.001). MCI also showed increased frontal delta during ripples (*p* = 0.002) and reduced frontal alpha 2 activity compared to Nold (*p* = 0.044). These differences highlight group‐specific alterations in vigilance‐related EEG rhythms, with ADMCI demonstrating pronounced frontal delta activity and reduced alpha reactivity.

**Conclusions:**

The findings suggest that AD and MCI patients show distinctive EEG rhythm disruptions during wakefulness and sleep transitions. AD is particularly characterized by frontal delta hyperactivity and impaired alpha reactivity, which may underlie their altered daytime vigilance.

**Significance:**

This study emphasizes the role of EEG biomarkers in differentiating cognitive impairment subtypes and understanding their unique impacts on vigilance and sleep transitions.

